# Deep learning model for measuring the sagittal Cobb angle on cervical spine computed tomography

**DOI:** 10.1186/s12880-023-01156-6

**Published:** 2023-11-28

**Authors:** Chunjie Wang, Ming Ni, Shuai Tian, Hanqiang Ouyang, Xiaoming Liu, Lianxi Fan, Pei Dong, Liang Jiang, Ning Lang, Huishu Yuan

**Affiliations:** 1 Department of Radiology, Peking University Third Hospital, 49 Huayuan North Road, Haidian District, Beijing, 100191 China; 2 Department of Orthopedics, Peking University Third Hospital, Beijing, 100191 China; 3 Engineering Research Center of Bone and Joint Precision Medicine, Beijing, 100191 China; 4 Beijing Key Laboratory of Spinal Disease Research, Beijing, 100191 China; 5 Beijing United Imaging Research Institute of Intelligent Imaging, Beijing, 100089 China; 6 United Imaging Intelligence (Beijing) Co., Ltd., Beijing, 100089 China

**Keywords:** Deep learning, Sagittal Cobb angle, Cervical spine, Computed tomography

## Abstract

**Purposes:**

To develop a deep learning (DL) model to measure the sagittal Cobb angle of the cervical spine on computed tomography (CT).

**Materials and methods:**

Two VB-Net-based DL models for cervical vertebra segmentation and key-point detection were developed. Four-points and line-fitting methods were used to calculate the sagittal Cobb angle automatically. The average value of the sagittal Cobb angle was manually measured by two doctors as the reference standard. The percentage of correct key points (PCK), matched samples t test, intraclass correlation coefficient (ICC), Pearson correlation coefficient, mean absolute error (MAE), and Bland‒Altman plots were used to evaluate the performance of the DL model and the robustness and generalization of the model on the external test set.

**Results:**

A total of 991 patients were included in the internal data set, and 112 patients were included in the external data set. The PCK of the DL model ranged from 78 to 100% in the test set. The four-points method, line-fitting method, and reference standard measured sagittal Cobb angles were − 1.10 ± 18.29°, 0.30 ± 13.36°, and 0.50 ± 12.83° in the internal test set and 4.55 ± 20.01°, 3.66 ± 18.55°, and 1.83 ± 12.02° in the external test set, respectively. The sagittal Cobb angle calculated by the four-points method and the line-fitting method maintained high consistency with the reference standard (internal test set: ICC = 0.75 and 0.97; r = 0.64 and 0.94; MAE = 5.42° and 3.23°, respectively; external test set: ICC = 0.74 and 0.80, r = 0.66 and 0.974, MAE = 5.25° and 4.68°, respectively).

**Conclusions:**

The DL model can accurately measure the sagittal Cobb angle of the cervical spine on CT. The line-fitting method shows a higher consistency with the doctors and a minor average absolute error.

## Introduction

Cervical lordosis is an important prerequisite for maintaining normal stability and flexibility of the cervical spine [1]. Loss of cervical lordosis is related to the occurrence and development of cervical degenerative diseases. By evaluating the degree of cervical lordosis, orthopedists can initially understand the severity of cervical degenerative diseases and carry out individualized treatment to improve the prognosis of patients [2, 3].

At present, the most commonly used method to evaluate cervical lordosis is the sagittal Cobb angle [4–7], which is the angle formed by the intersection of the C2 and C7 inferior endplate extension lines, and Dr. John R. Cobb first described it in 1948 [8]. Clinically, the sagittal Cobb angle is mainly determined by manual measurements. Previous study have show that the average Cobb angle measured by X-ray in normal people is 9.76° [9], and the average Cobb angle based on CT measurements is 12.60°±7.78° (mean ± standard deviation) [10]. However, the manual measurement of the sagittal Cobb angle has the disadvantages of being time-consuming, having strong subjectivity and large measurement errors [11, 12]. As a result, the current measurement results of the Cobb angle still have inter-individual differences and limit the versatility of the Cobb angle. Although the Cobb angle measurement can be completed on the cervical spine X-ray, it is also important to evaluate the cervical spine curvature when the patient undergoes a CT examination, especially during preoperative planning. At the same time, since it is easier to observe the lower edge of the C7 vertebral body in CT of the cervical spine than in X-ray, the Cobb angle was measured on CT images in this study.

As research on deep learning (DL) continues to increase, it has been widely used in tasks such as disease identification, classification, and diagnosis, and some studies have proven that the performance of DL is comparable to that of experienced radiologists [13–15]. Recent studies have used DL for quantitative measurement tasks in radiology [16, 17]. The research results showed that the use of DL for automatic quantitative measurement can improve the consistency and objectivity of the measurement results and save time.

Therefore, we aimed to propose an automatic quantitative measurement method of the sagittal Cobb angle on computed tomography (CT) with a DL model as the core, evaluate the performance of the model by comparing it with the reference standard measurement value in an internal test set, and evaluate the robustness and generalization of the model in an external test set.

## Materials and methods

This retrospective study was approved by the Medical Science Research Ethics Committee of our hospital, and the requirement for informed consent was waived.

### Data set preparation

**Fig. 1:**
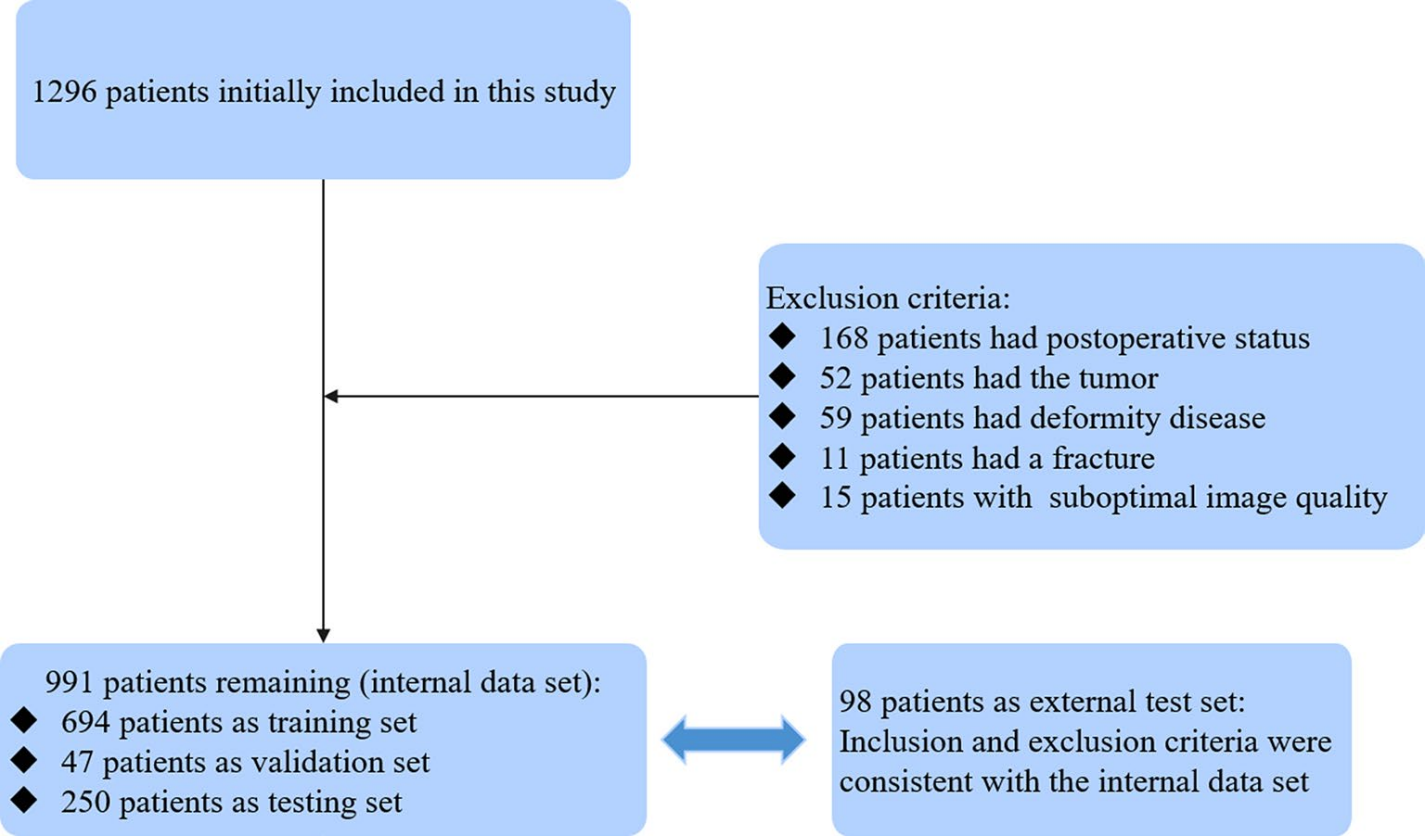
The detailed process of enrolling patients in this study

The images of patients who underwent cervical CT at our hospital from April 2017 to August 2017 were retrospectively collected as the internal data set. The inclusion criterion was age ≥ 18 years. The exclusion criteria were as follows: (1) patients who underwent cervical spine surgery before the CT examination; (2) patients with cervical spine deformities, fractures, infections, tumors, or other reasons leading to an unclear display of the vertebral anatomy; and (3) poor image quality and incomplete scan data. After the enrolled patients were determined, 70% of the data set was used as the training set, 5% was used as the validation set, and the remaining 25% was used as the test set. The external test set consisted of images of cervical spine CT scans obtained from two other medical centers from January 2021 to March 2021, and the inclusion and exclusion criteria were consistent with those for the internal data set. The patient enrollment process is shown in Fig. 1.

### CT scanning

The internal data set images were collected from Siemens Definition Flash CT (Erlangen, Germany; n = 585) and GE Healthcare Discovery CT750 (Milwaukee, Wisconsin, USA; n = 406) scanners, and the external test set images were collected from Siemens Go-Top (Forchheim, Germany; n = 51) and GE Healthcare Optima CT620 (Milwaukee, Wisconsin, USA; n = 47) scanners. All scans used the automatic tube current function (range is 0-500mAs), and the corresponding parameters were automatically set according to the patient’s height, weight, etc. Table 1 provides detailed information on the different CT scanners and the scan parameters.

**Table 1:** Parameters for cervical spine CT

CT parameters	Siemens (Definition Flash CT)	GE (Discovery CT750)	GE (Optima CT680)	Siemens (Go-Top)
Acquisition mode Tube voltage (kV)	Helical 120	Helical 120	Helical 120	Helical 120
Tube current (mA)	CARE Dose 4D	Smart mA	Smart mA	CARE Dose 4D
Pitch	0.6	0.984	0.984	0.6
Thickness/Increment (mm)	1.0/1.0	1.25/1.25	1.25/1.25	1.0/1.0
Recon thickness (sagittal mm)	3	3	3	3

### Data set labeling

**Fig. 2:**
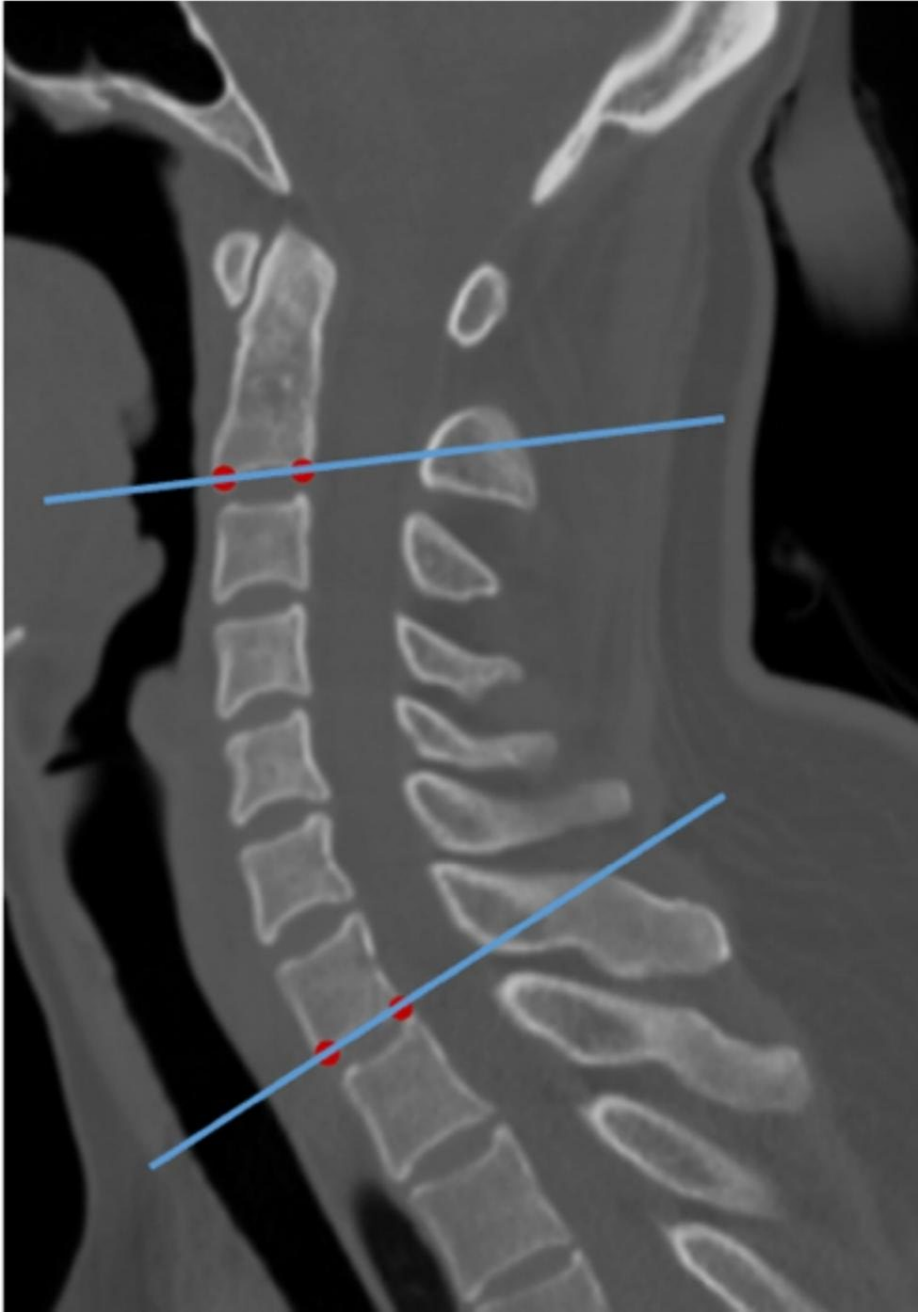
An example of the doctor labeling the key points of the lower edges of the C2 and C7 vertebrae in the midsagittal image

Three doctors (doctor 1, 6 years of experience; doctor 2, 3 years of experience; and doctor 3, 30 years of experience) labeled the key points and measured the sagittal Cobb angle at the mid-sagittal level, as shown in Fig. 2.

### Labeling of the key points

The vertebral body segmentation algorithm of VB-Net (an algorithm developed earlier by this research group) was used to calculate the area of the C2 vertebral body in each image in the sagittal image, and the image with the largest C2 area was selected as the midsagittal position. Doctors 1 and 2 independently labeled the key points in all the data sets. In the training set, the distance between the labeled points of the two doctors at the same position was calculated, and the outlier of the distance between the labeled points (Tp) was calculated. In the internal and external test sets, if the distance between the two doctors’ labeling points at the same position was less than Tp, the labeled point was considered reliable; otherwise, the labeled point was considered unreliable and needed to be corrected. The outlier formula [18] was as follows:

$${\rm{Tp = Q3 + 1}}{\rm{.5 \times }}\left( {{\rm{Q3}} - {\rm{Q1}}} \right)$$

Q1 and Q3: the lower and upper quartiles of the distance between the two doctors’ markings of the same key point.

To correct unreliable cases, doctor 3 independently labeled unreliable cases. The distances between the labeled points of doctors 1, 2, and 3 were calculated. The annotation results of the two doctors with the smallest distance were used as the final standard. After the calculation, the internal and external test sets were revised. For the same location, the midpoint of the points marked by the two doctors was used as the final key point for model development and testing.

### Measurement of the sagittal Cobb angle

In the test set, doctor 1 and doctor 2 independently measured the sagittal Cobb angle at the midsagittal level. The absolute difference in the sagittal Cobb angle of the same case measured by the two doctors was calculated, and the outlier of the absolute difference (Tcobb) was calculated. If the absolute difference in the sagittal Cobb angle of the same case measured by two doctors was less than Tcobb, the case was considered reliable, and the average value of the two measurements was used in the final data; otherwise, the measurement was considered unreliable, and doctor 3 made corrections.

## Deep learning workflow

**Fig. 3:**
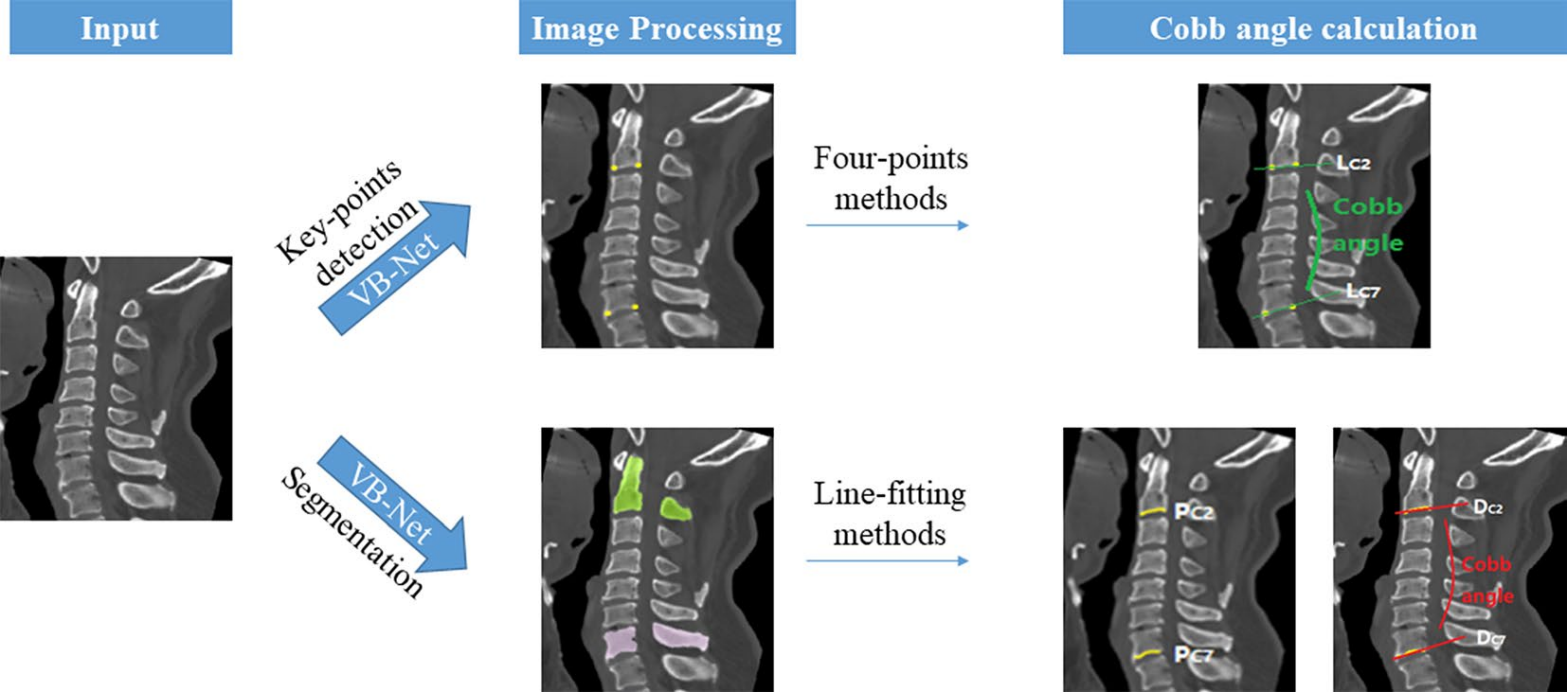
Workflow of the deep learning method to measure the sagittal Cobb angle

**Fig. 4:**
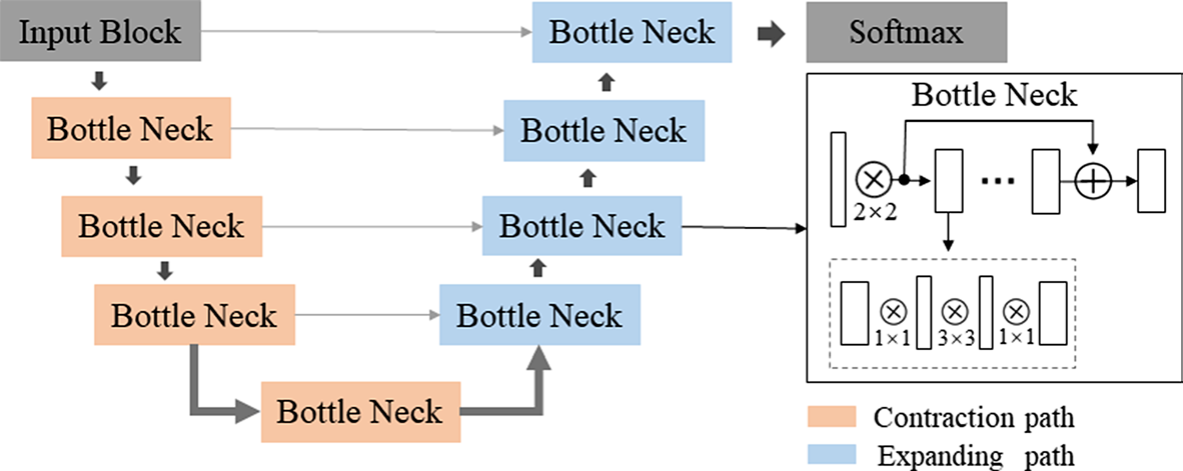
Network architecture of VB-Net. In VB-Net, a bottle-neck structure replaces the conventional layers in both the decoder and encoder of traditional U-Net. VB-Net can largely decrease the model size and promote accuracy

The deep learning algorithm was developed on PyTorch with an NVIDIA GeForce GTX TITAN X graphics card (video memory 12 GB). The workflow of the DL model used to measure the sagittal Cobb angle is shown in Fig. 3. This study developed two different VB-Net-based DL models for cervical vertebra segmentation and key-point detection. The architecture of VB-Net is shown in Fig. 4. The VB-Net consists of bottleneck blocks and a V-Net [19].

This study used a multilabel VB-Net to segment the cervical vertebrae on CT images. To detect the background area, bottom anterior corners, and bottom posterior corners in the C2 and C7 vertebra, we set the field of view and output channel as 2.4 mm × 2.4 mm and 5 for key point detection in another VB-Net. The key points were detected by the four-points and line-fitting methods. The details of the two methods are as follows:

1. Four-points method: We connected the anterior and posterior corners of the lower edge of the C2 and C7 vertebral bodies detected by the key-point detection model into two line segments, called LC2 and LC7, and the angle between LC2 and LC7 was the estimated sagittal Cobb angle in the four-points method.
2. Line-fitting method: The cervical vertebra segmentation model segments the lower edge of the C2 and C7 vertebral bodies, and the anterior and posterior corners of the lower edge of the C2 and C7 vertebral bodies detected by the key-point detection model are used as reference points to extract a series of point sets PC2 and PC7 on the lower edge of the C2 and C7 vertebral bodies. Finally, we used the least squares method to fit DC2 and DC7 on PC2 and PC7, respectively; the angle between DC2 and DC7 was the estimated sagittal Cobb angle in the line-fitting method.

## Statistical analysis

The percentage of correct key points (PCK) [20, 21] was used to evaluate the performance of the key point detection of the model. For different key point targets, if the distance between the key points automatically detected by the model and the manually marked key points was less than the key point outlier, the key point model detection was considered correct. The PCK was the percentage of key points predicted correctly for all predicted key points. The matched samples t test was used to compare whether there were significant differences between the sagittal Cobb angle measured by the four-points method, line-fitting method, and reference standard. When the p value was > 0.05, there was no significant difference between the two groups of samples tested. The intraclass correlation coefficient (ICC) and Pearson correlation coefficient (r) were used to evaluate the consistency and correlation of the DL model and the reference standard to measure the sagittal Cobb angle. ICC < 0.4 indicated poor consistency, 0.4≤ICC < 0.6 indicated general consistency, 0.6≤ICC < 0.75 indicated good consistency, and ICC ≥0.75 indicated excellent consistency. |r| <0.4 indicated a weak correlation, 0.6≤|r|<0.8 indicated a strong correlation, and |r| ≥0.80 indicated an extremely strong correlation. The average absolute error was used to evaluate the measurement error between the model and the reference standard: mean absolute error (MAE)=$\frac{1}{n}\sum _{i=1}^{n}\left|\text{o}\text{b}\text{s}\text{e}\text{r}\text{v}\text{e}\text{d}\right(\text{i})-\text{p}\text{r}\text{e}\text{d}\text{i}\text{c}\text{t}\text{e}\text{d}(\text{i}\left)\right|$. Finally, Bland‒Altman plots were created to show the mean difference, standard deviation, and 95% limit of agreement (LoA) between the reference standard and model estimate measurements. SPSS version 24 (IBM Corp., Chicago, Illinois, USA) was used to perform all statistical analyses.

## Results

### Patient characteristics by data set

In total, 991 patients were included in the internal data set, of whom 694 were in the training set (mean age, 52 ± 19 years; range, 25–89 years), comprising 422 men (mean age, 55 ± 10 years; range, 21–89 years) and 272 women (average age, 54 ± 10 years; range, 20–76 years); 47 were in the training set (mean age, 52 ± 13 years; range, 20–89 years), comprising 28 men (mean age, 49 ± 16 years; range, 20–89 years) and 19 women (average age, 54 ± 10 years; range, 20–79 years); and 250 patients were in the testing set (mean age, 53 ± 10 years; range, 20–80 years), comprising 146 men (mean age, 53 ± 10 years; range, 23–80 years) and 104 women (mean age, 54 ± 10 years; range, 20–76 years). A total of 98 cases were screened for the external test set, comprising 59 men (mean age, 50 ± 11 years; range, 27–74 years) and 39 women (mean age, 57 ± 11 years; age range, 32–79 years).

### Outliers of key points and sagittal Cobb angle measurements

According to the outlier formula, the outlier of the anterior corner of the lower edge of the C2 vertebral body was 2.8 mm, the outlier of the posterior corner of the lower edge of the C2 vertebral body was 2.7 mm, the outlier of the anterior corner of the lower edge of the C7 vertebral body was 2.7 mm, and the outlier of the posterior corner of the lower edge of the C7 vertebral body was 2.8 mm. The Tcobb angle was 5.37°.

### Performance of key point detection

The PKC range of the anterior and posterior corners of the lower edges of C2 and C7 in the internal test set was 98.4 − 100%, and the PKC range of the f anterior and posterior corners of the lower edges of C2 and C7 in the external test set was 78.0 − 85.0%. Table 2 shows the PCK values of the anterior and posterior corners of the lower edges of C2 and C7 in the internal and external test sets.

**Table 2:** Data of the percentage of correct key points in the test set

	Anterior corner of the C2 lower edge	Posterior corner of the C2 lower edge	Anterior corner of the C7 lower edge	Posterior corner of the C7 lower edge
Outlier (mm)	2.8	2.7	2.7	2.8
Internal test set	100%	99.6%	98.4%	99.2%
External test set	85.0%	84.0%	78.0%	80.0%

### Performance of the sagittal Cobb angle measurements

In the internal test set, the four-points method, line-fitting method, and reference standard measured sagittal Cobb angles were − 1.10 ± 18.29°, 0.30 ± 13.36°, and 0.50 ± 12.83°, respectively. In the external test set, the four-points method, line-fitting method, and reference standard measured sagittal Cobb angles were 4.55 ± 20.01°, 3.66 ± 18.55°, and 1.83 ± 12.02°, respectively. There was no significant difference between the model and reference standard measured values (P > 0.05) (Table 3).

**Table 3:** Measurement results of the sagittal Cobb angle in the test set

	Reference standard	Four-points method	P-value	t	Line-fitting method	P-value	t
Internal test set	0.50 ± 12.83°	-1.10 ± 18.29°,	0.05	-0.95	0.30 ± 13.36°	0.72	-0.36
External test set	1.83 ± 12.02°	4.55 ± 20.01°	0.16	-1.43	3.66 ± 18.55°	0.08	-1.78

**Fig. 5:**
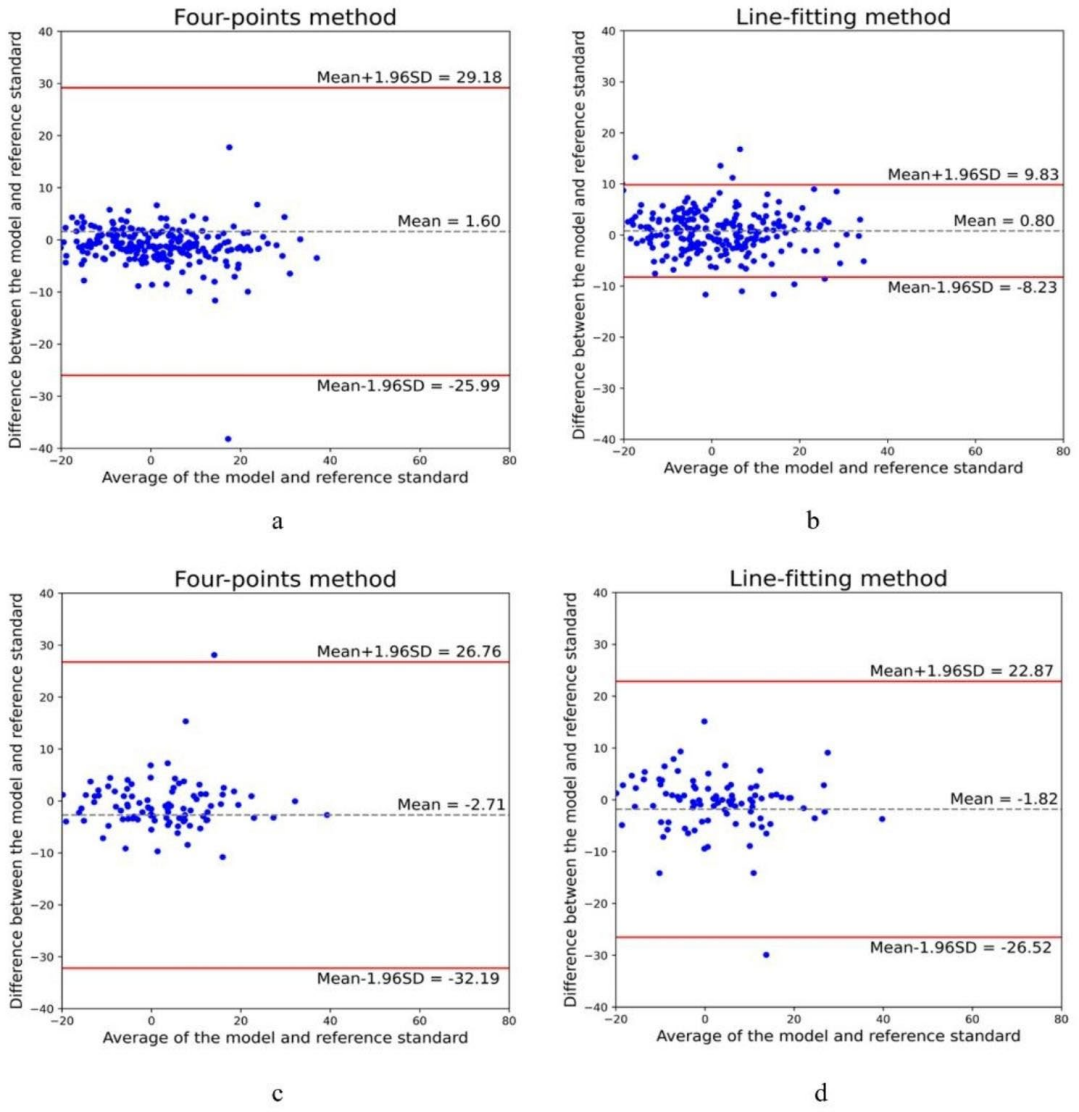
Bland–Altman plots showing differences between the model and reference standard over the range of the mean of both. (**a**) Four-points method’s Bland–Altman plots for the internal test set. (**b**) Line-fitting method’s Bland–Altman plots for the internal test set. (**c**) Bland–Altman plots for the four-points method for the external test set. (**d**) Line-fitting method’s Bland–Altman plots for the external test set

**Fig. 6:**
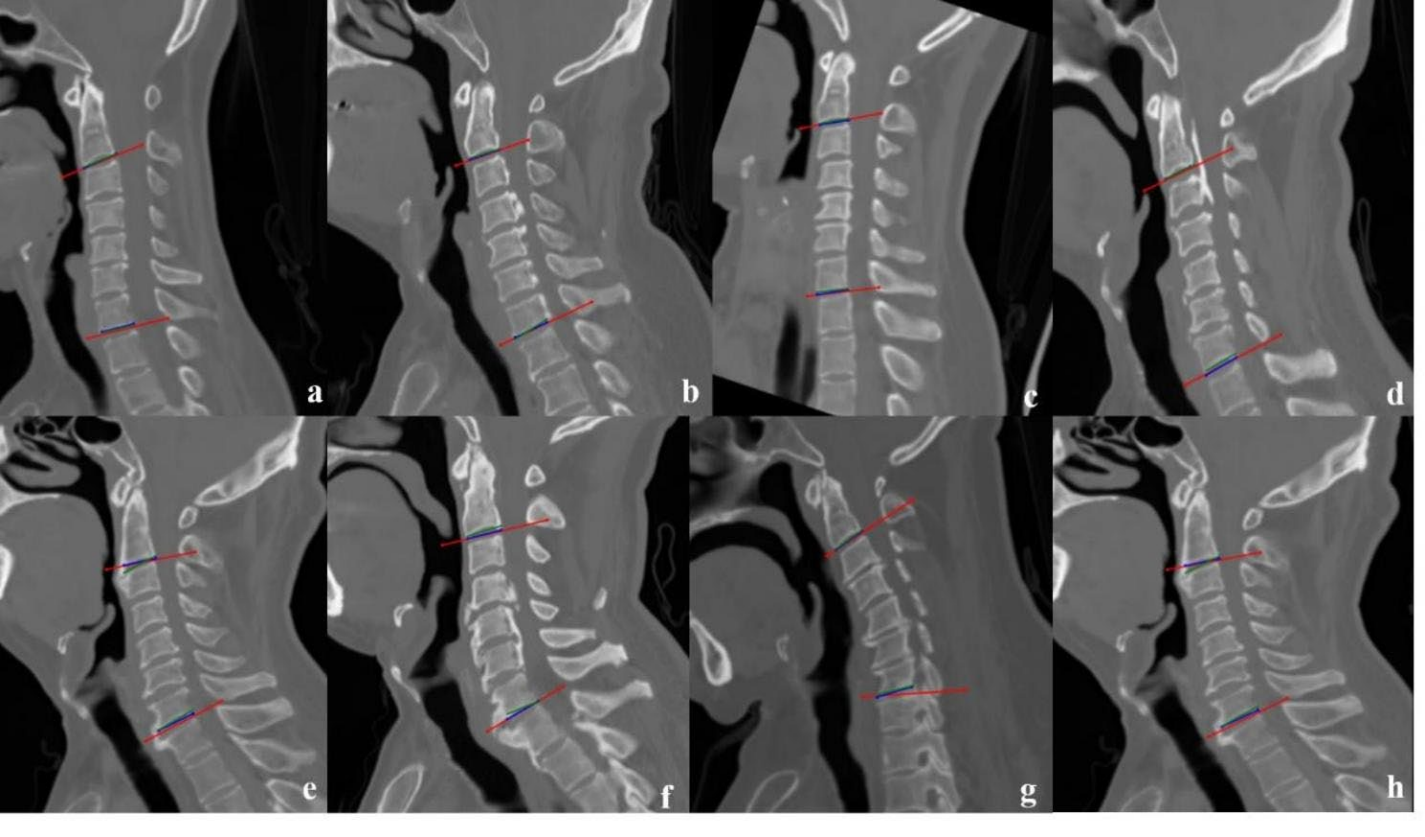
The example of doctors and VB-Net marked the Cobb angle measurement results using the line-fitting method. The green and blue lines represent the final results marked by the two doctors, and the red line is the result of VB-Net. The cases in Figures **a-d** are highly consistent with the doctors’ standard, and good results can still be obtained in the presence of vertebral osteophytes and ossification of the posterior longitudinal ligament. Figures **e-h** show the cases where there is a significant difference between VB-Net and manual measurement, mainly due to bone hyperplasia, sclerosis, and osteophytes

On further comparing the overall performance of the model with the reference standard, precise measurements were found for the three parameters (internal test set: for the four-points and line-fitting methods, ICC = 0.75 and 0.97, r = 0.64 and 0.94, MAE = 3.23 and 5.42, respectively; external test set: for the four-points and line-fitting methods, ICC = 0.74 and 0.80, r = 0.74 and 0.80, MAE = 4.68 and 5.25, respectively) (Table 4). The mean difference and 95% LoA in the internal and external test sets are shown in the Bland–Altman plots (Fig. 5a–d). The Cobb angle measured by the line-fitting method by the doctor and DL is shown in Fig. 6.

**Table 4:** Comparison of model estimates and the reference standard of the three parameters in the test set

	Method	ICC	r	MAE (°)
Internal test set	Four-points method	0.75 (0.68, 0.81)	0.64 (0,49,0.81)	5.42 (3.96,7.26)
	Line-fitting method	0.97 (0.96, 0.98)	0.94 (0.91,0.97)	3.23 (2.84,3.69)
External test set	Four-points method	0.74 (0.60,0.82)	0.66 (0.55,0.95)	5.25 (3.01,8.58)
	Line-fitting method	0.80 (0.71,0.87)	0.74 (0.66,0.94)	4.68 (3.00,7.40)

Data in parentheses are 95% confidence intervals

ICC, intraclass correlation coefficient; MAE, mean absolute error

In addition, we analysed the time it took to use the four-points and line-fitting methods by DL. It took 48 ms and 51 ms to complete the sagittal Cobb angle measurement for one patient.

## Discussion

In this study, we developed DL models to accurately measure the sagittal Cobb angle of the cervical spine on CT, and the line-fitting method showed a higher consistency with the doctors and a minor average absolute error.

Cervical degenerative disease is one of the most common causes of neck pain and is the fourth leading contributor to disability-adjusted life years globally, with a significant increase in prevalence and disability that is likely to increase with further population aging [22, 23]. The early stage of cervical degenerative diseases is usually accompanied by the disappearance of the physiological curvature of the cervical spine. Clinically, by measuring the sagittal Cobb angle of the cervical spine, we can understand the severity of cervical degenerative diseases, assist in formulating appropriate treatment plans early, and help prevent the further development of the disease [3, 24]. Due to the increasing number of patients, manual measurement of the sagittal Cobb angle of the cervical spine is time-consuming and highly subjective, which makes it impossible to routinely report the sagittal Cobb angle of the cervical spine in the daily work of the radiology department. The use of DL for quantitative measurement tasks can compensate for the shortcomings of manual measurement of the sagittal Cobb angle to a certain extent.

Although current research suggests that CT-based Cobb angle measurement may not be as accurate as X-ray, mainly caused by the patient positioning in image acquisition, assessment of cervical spine curvature is still necessary when performing cervical spine CT reporting. When cervical spine surgery is needed, measuring the Cobb angle and evaluating the soft tissue condition in CT images simultaneously can help comprehensively assess the patient’s spinal condition before surgery and help determine the development of surgical plans. Equally important, CT-based Cobb angle measurement can also be used as part of the artificial intelligence structured report of cervical spine CT.

Calculating the PCK is necessary to measure the sagittal Cobb angle using the DL model. The DL model used to measure the sagittal Cobb angle was mainly based on the straight line formed by the key points of the model detection. When the two key points forming the straight line have large deviations from the true value and the trend is consistent, the straight line formed by the key points may have no obvious angle difference between the upper or lower edge of the vertebral body, so there may be a situation where the key point detection results of the DL model are not good but the measured value of the sagittal Cobb angle is highly consistent with the reference standard. Therefore, when using DL to conduct similar quantitative measurement research based on key point detection and statistical analysis of the final measurement indicators, the key point detection results should also be reported.

This study explored the application of the four-points method and the line-fitting method to measure the sagittal Cobb angle. Before measuring the COBB angle, it is essential to select a reasonable image for measurement. After segmenting the cervical vertebrae, the image with the largest C2 vertebral body area was selected as the midsagittal position for model training and verification. This method helps reduce measurement errors caused by differences in the manual selection of images. For the sagittal Cobb angle measurement, we found that the line-fitting method has higher consistency and correlation than the four-points method, and the overall performance of the line-fitting method is better than that of the four-pointsmethod. This situation may be because the lower edge of the vertebral body is not a straight line on the cervical CT image. The line-fitting method extracts a series of points on the lower edge of the vertebral body and fits the vertebral body using the least-squares method. Compared with the four-points method, which directly connects the anterior and posterior corners of the lower edge of the vertebral body, a certain error is reduced; thus, the performance of the line-fitting method is better than that of the four-points method.

At present, the measurement of the Cobb angle has received increasing attention. Current DL research on the Cobb angle is mainly based on the full length of the spine or the thoracolumbar segment. At the same time, there are relatively few studies on the Cobb angle of the cervical spine, mainly focusing on X-ray images [25–27]. Wang et al. [28] developed a deep learning model integrating anteroposterior and lateral X-rays to measure the thoracic Cobb angle, and the MAE of the measured Cobb angle reached 6.26°- 7.81°. Sun et al. [17] developed a DL model based on key point detection to measure the Cobb angle of scoliosis, and the Cobb angle measured by the model had high consistency and correlation with human experts (ICC = 0.994, r = 0.984). Our research focuses on the development of a high-precision and fast measurement DL model of the Cobb angle based on cervical spine CT. Compared with previous studies, the DL model we developed for Cobb angle measurement has smaller errors and achieves a high degree of consistency with doctors.

## Limitations

[1] The osteophyte at the edge of the vertebral body or the ossification of the anterior longitudinal ligament or the posterior longitudinal ligament may affect the accuracy of vertebral body segmentation and key point detection [2]. The study did not include minors, cervical spine surgery, cervical spine fractures, or deformities. The model currently cannot automatically measure the Cobb angle for cervical spine CT in this type of patient [3]. This study only studied the measurement of the Cobb angle of the cervical spine. It did not comprehensively evaluate cervical spine degenerative diseases, nor did it determine normal/abnormal Cobb angle values.

## Conclusions

The DL model can accurately measure the sagittal Cobb angle on CT. Compared with the four-points method, the line-fitting method shows a higher consistency with the doctors and a minor average absolute error. The deep learning method developed in our study is a potential tool to help radiologists and clinicians quantitatively evaluate cervical degenerative diseases.

## Data Availability

Data generated or analysed during the study are available from the corresponding author by request.

## Abbreviations

DL: deep learning
CT: computed tomography
TP: distance between labeled points
Tcobb: outlier of the absolute difference
PCL: percentage of correct key points
ICC: intraclass correlation coefficient
MAE: mean absolute error

## References

[1] Scheer JK, Tang JA, Smith JS (2013). Cervical spine alignment, sagittal deformity, and clinical implications: a review [J]. J Neurosurg Spine.

[2] Miyazaki M, Hymanson HJ, Morishita Y (2008). Kinematic analysis of the relationship between sagittal alignment and disc degeneration in the cervical spine [J]. Spine.

[3] Tang JA, Scheer JK, Smith JS (2015). The impact of standing regional cervical sagittal alignment on outcomes in posterior cervical fusion Surgery [J]. Neurosurgery.

[4] Ames CP, Blondel B, Scheer JK (2013). Cervical radiographical alignment: comprehensive assessment techniques and potential importance in cervical myelopathy [J]. Spine.

[5] Polly DW, Kilkelly FX, McHale KA, Asplund LM, Mulligan M, Chang AS (1996). Measurement of lumbar lordosis - evaluation of intraobserver, interobserver, and technique variability [J]. Spine.

[6] Singer KP, Jones TJ, Breidahl PD (1990). A comparison of Radiographic and Computer-assisted measurements of thoracic and Thoracolumbar Sagittal curvature [J]. Skeletal Radiol.

[7] Polly DW Jr., Kilkelly FX, McHale KA, Asplund LM, Mulligan M, Chang AS. Measurement of lumbar lordosis. Evaluation of intraobserver, interobserver, and technique variability [J]. Spine, 1996, 21(13): 1530-5; discussion 5–6. 10.1097/00007632-199607010-00008 8817780

[8] Cobb JJ, A A o O. S. Outline for the study of scoliosis. Instructional course lectures [J]. 1948, 5(.

[9] Park SM, Song KS, Park SH, Kang H, Daniel Riew K (2015). Does whole-spine lateral radiograph with clavicle positioning reflect the correct cervical sagittal alignment?. Eur Spine Journal: Official Publication Eur Spine Soc Eur Spinal Deformity Soc Eur Sect Cerv Spine Res Soc.

[10] Park JH, Cho CB, Song JH, Kim SW, Ha Y, Oh JK (2013). T1 slope and cervical sagittal alignment on cervical CT radiographs of asymptomatic persons [J]. J Korean Neurosurg Soc.

[11] Harrison DE, Harrison DD, Cailliet R, Janik TJ, Holland B (2001). Radiographic analysis of lumbar lordosis: centroid, Cobb, TRALL, and Harrison posterior tangent methods [J]. Spine.

[12] Harrison DE, Harrison DD, Cailliet R, Troyanovich SJ, Janik TJ, Holland B (2000). Cobb method or Harrison posterior tangent method: which to choose for lateral cervical radiographic analysis [J]. Spine.

[13] Esteva A, Robicquet A, Ramsundar B (2019). A guide to deep learning in healthcare [J]. Nat Med.

[14] Hallinan J, Zhu L, Yang K (2021). Deep learning model for automated detection and classification of Central Canal, lateral recess, and neural Foraminal stenosis at lumbar spine MRI [J]. Radiology.

[15] Montagnon E, Cerny M, Cadrin-Chênevert A (2020). Deep learning workflow in radiology: a primer [J]. Insights into Imaging.

[16] Fischer M, Walter SS, Hepp T (2021). Automated morphometric analysis of the Hip Joint on MRI from the German National Cohort Study [J]. Radiol Artif Intell.

[17] Sun Y, Xing Y, Zhao Z, Meng X, Xu G, Hai Y. Comparison of manual versus automated measurement of Cobb angle in idiopathic scoliosis based on a deep learning keypoint detection technology [J]. European spine journal: official publication of the European Spine Society, the European Spinal Deformity Society, and the European section of the Cervical Spine Research Society, 2022, 31(8): 1969–78. 10.1007/s00586-021-07025-6 34716822

[18] Lindley DV, J T M G. Introduction to the practice of statistics, (3rd edition), by Moore David S. and, McCabe George P. Pp. 825 (with appendices and CD-ROM). ï¿¡27.95. 1999. ISBN 0 7167 3502 4 (W. H. Freeman) [J]. 1999, 83(497): 825 – 375.

[19] Milletari F, Navab N, Ahmadi SA. V-Net: Fully Convolutional Neural Networks for Volumetric Medical Image Segmentation; proceedings of the 2016 Fourth International Conference on 3D Vision (3DV), F, 2016 [C].

[20] Bier B, Goldmann F, Zaech JN (2019). Learning to detect anatomical landmarks of the pelvis in X-rays from arbitrary views [J]. Int J Comput Assist Radiol Surg.

[21] Chen HC, Lin CJ, Wu CH, Wang CK, Sun YN (2010). Automatic Insall-Salvati ratio measurement on lateral knee x-ray images using model-guided landmark localization [J]. Phys Med Biol.

[22] Theodore N (2020). Degenerative cervical spondylosis [J]. N Engl J Med.

[23] Hurwitz EL, Randhawa K, Yu H, Côté P, Haldeman S. The Global Spine Care Initiative: a summary of the global burden of low back and neck pain studies [J]. European spine journal: official publication of the European Spine Society, the European Spinal Deformity Society, and the European Section of the Cervical Spine Research Society, 2018, 27(Suppl 6): 796–801. 10.1007/s00586-017-5432-9 29480409

[24] Suk KS, Kim KT, Lee JH, Lee SH, Lim YJ, Kim JS (2007). Sagittal alignment of the cervical spine after the laminoplasty [J]. Spine.

[25] Gami P, Qiu K, Kannappan S et al. Semiautomated intraoperative measurement of Cobb angle and coronal C7 plumb line using deep learning and computer vision for scoliosis correction: a feasibility study [J]. J Neurosurg Spine, 2022, 1–9. 10.3171/2022.4.SPINE22133 36303475

[26] Jin C, Wang S, Yang G, Li E, Liang Z. A review of the methods on Cobb Angle Measurements for spinal curvature [J]. Sens (Basel), 2022, 22(9). 10.3390/s22093258 PMC9101880 35590951

[27] Alukaev D, Kiselev S, Mustafaev T, Ainur A, Ibragimov B, Vrtovec T. A deep learning framework for vertebral morphometry and Cobb angle measurement with external validation [J]. European spine journal: official publication of the European Spine Society, the European Spinal Deformity Society, and the European section of the Cervical Spine Research Society, 2022, 31(8): 2115–24. 10.1007/s00586-022-07245-4 35596800

[28] Wang L, Xu Q, Leung S, Chung J, Chen B, Li S. Accurate automated Cobb angles estimation using multi-view extrapolation net [J]. Medical image analysis, 2019, 58(101542. 10.1016/j.media.2019.101542 31473518

